# miR-107-enriched exosomes promote ROS/wnt/autophagy, inhibit intracellular mycobacterial growth and attenuate lung infection

**DOI:** 10.3389/fimmu.2025.1567167

**Published:** 2025-07-04

**Authors:** Wei Xu, Yuan Wu, Min Yang, Jiayu Zhou, Liying Zhu, Xiaosai Ma, Chonghai Qiu, Ling Shen, Hongbo Shen, Feifei Wang

**Affiliations:** ^1^ Key Laboratory of Medical Molecular Virology (MOE/NHC/CAMS), Shanghai Institute of Infectious Disease and Biosecurity, Department of Medical Microbiology and Parasitology, School of Basic Medical Sciences, Shanghai Medical College, Fudan University, Shanghai, China; ^2^ Department of Microbiology & Immunology and Center for Primate Biomedical Research, University of Illinois College of Medicine, Chicago, IL, United States; ^3^ Shanghai Clinical Research Center for Infectious Disease (tuberculosis), Shanghai Key Laboratory of Tuberculosis, Shanghai Pulmonary Hospital, Key Laboratory of Pathogen-Host Interaction Ministry of Education, Institute for Advanced Study, Tongji University School of Medicine, Shanghai, China; ^4^ Shanghai Sci-Tech Inno Center for Infection & Immunity, Shanghai, China

**Keywords:** mycobacteria tuberculosis, plasma exosomes, miR-107, ROS, Wnt

## Abstract

Exosomes, known as small membrane vesicles of endocytic origin produced by most cell types, exist in a variety of body fluids including plasma. The roles of exosomes in immune responses against *Mycobacteria tuberculosis* (*Mtb*) infection remain poorly characterized. Here, we found that miR-107 highly expressed in exosomes from plasma of TB patients but not healthy control (HC) subjects. Consistently, such miR-107-high exosomes were also detected in both the extracellular fluid released by mycobacterial-infected macrophages and the plasma of mycobacterial-infected mice. Interestingly, adding the miR-107-high plasma exosomes or the miR-107 mimics to infected THP-1 macrophages inhibited intracellular mycobacterial growth. Consistently, while nanoscale and fluorescence imaging revealed that miR-107 could be transferred inter-cellularly via exosomes, miR-107-enriched exosomes from miR-107 overexpressing cells also inhibited mycobacterial growth in THP-1 macrophages and primary monocytes/peripheral blood mononuclear cells (PBMC). Mechanistically, miR-107-high exosomes increased ROS production; miR-107 regulated Wnt pathway by targeting Wnt16 and promoted autophagy in THP-1 macrophages. Furthermore, treatment of infected mice with miR-107-enriched exosomes reduced mycobacterial infection in lung tissues. Our results raise a possibility to explore miR-107-high plasma exosomes for a potential surrogate marker for TB. Findings suggest that exosomes enriched with miR-107 or other bio-active molecules may potentially serve as an attractive approach for treatment of infection.

## 1 Introduction

After pandemic of COVID-19, tuberculosis (TB) caused by *Mycobacterium tuberculosis* (*Mtb*) returned as the top cause for infectious disease-related deaths in 2023. Eradication of TB faces many challenges including limited access to diagnosis and treatment, drug-resistant TB (1). Thus, there is urgent need to develop novel effective diagnostic and treatment methods based on the in-depth understanding of TB and the host responses. The balance between immune response and TB infection appears to determine which one of potential clinical outcomes of active TB, latent TB infection (LTBI) or resister status with early clearance of TB bacillus without immune signature of infection (2, 3).

Exosomes, classified as nanoscale sizes (30–150 nm), are phospholipid bilayer extracellular vehicles generated by all host cells (4). Exosomes, evolutionarily conserved mediators of intercellular communication, transport a diverse array of bioactive molecules—including nucleic acids, proteins, and lipids—that critically influence pathophysiological processes in both chronic and acute disease states (5).In oncological contexts, tumor-derived exosomes actively shuttle multiple oncogenic cargos that drive malignant progression through mechanisms encompassing neoplastic proliferation, angiogenic induction, and immune system subversion. Notably, exosomal circSAFB2 facilitates M2 macrophage polarization in renal cell carcinoma via the miR-620/JAK1/STAT3 signaling axis, thereby establishing an immunosuppressive tumor microenvironment that potentiates metastatic dissemination (6). Similarly, elevated serum levels of exosomal circSHKBP1 in gastric cancer patients demonstrate significant correlations with vascular invasion and advanced TNM staging, suggesting its potential as a prognostic biomarker (7). Within neurodegenerative pathology, neuron- and glia-derived exosomes have emerged as critical vectors for intercellular transfer of pathogenic proteins in Alzheimer’s and Parkinson’s diseases, while simultaneously offering diagnostic potential through their detectable presence in biofluids (8).

The functional dichotomy of exosomes in microbial pathogenesis warrants particular attention. These vesicles exhibit dual functionality as both immune-modulatory agents and pathogen-exploited delivery systems. Post-bacterial invasion, host-derived exosomes incorporate microbial antigens that may paradoxically attenuate proinflammatory responses through suppression of cytokine secretion, induction of anti-inflammatory mediators, or targeted immune cell apoptosis (9). Concurrently, bacterially secreted exosomes transport virulence determinants capable of hijacking host cellular machinery, precipitating inflammatory cascades and cytotoxic effects upon recipient cell internalization (10). *Mycobacterium tuberculosis* infection presents unique challenges due to its exceptional transmissibility, protracted multidrug treatment requirements, and absence of universally effective vaccines. This critical unmet medical need underscores the imperative to develop novel exosome-based diagnostic platforms and targeted therapeutic interventions for improved tuberculosis management.

During *Mtb* infection, exosomes might potentially serve as diagnostic biomarkers since exosomes display distinctive profiles and quantities, with a revealing glimpse into the evolving landscape of *Mtb* infections (11). Exosomal-based biomarkers are gaining some interests in TB diagnostic since they exist in a variety of body fluids including bronchoalveolar lavage fluid, blood, saliva, breast milk, sweat, urine, and plasma (12, 13). Moreover, some exosomes, such as ones secreted by mesenchymal stem cells, have potential to be vaccine platforms and drug delivery vehicles for TB treatment (14). The diversified functions of exosomes were mainly determined by their components. Further studies are needed to understand functions and components of exosomes in the context of *Mtb* infection and TB pathogenesis.

To further investigate the roles of exosomes in immune responses induced by *Mtb* infection, we have isolated exosomes from plasma of TB patients and healthy control (HC) subjects and compared their miRNA expression profiles, their biological functions and immune regulation. We demonstrated that miR-107 highly expressed in exosomes in plasma of TB patients but not HC, and miR-107-enriched exosomes could potently inhibit mycobacterial growth in macrophages. Furthermore, treatment of infected mice with miR-107-enriched exosomes reduced mycobacterial infection levels and histology changes in lung tissues.

## 2 Materials and methods

### 2.1 Ethics statement

This study was approved by the institutional review boards for human subject research and the institutional biosafety committees at Shanghai Pulmonary Hospital (SPH) of Tongji University (approval number: K22-143Z). All subjects were adults and provided written informed consent. TB patients and HC were recruited from SPH. All procedures were performed in accordance with the protocol approved by the Ethics Committee of Shanghai Pulmonary Hospital of Tongji University. Patients were included in the study according to the following criteria: (1) evidence of active tuberculosis based on routine tuberculosis diagnostic tests, including the chest imaging and the T-SPOT.TB test; (2) patients had never been treated for TB or the anti-TB treatment duration was within 1 week. The exclusion criteria of patients were as follows:(1) patients with an obscure diagnosis or with other infectious diseases (hepatitis B or human immunodeficiency virus); (2) patients with severe hepatic or renal dysfunction; (3) and patients with positive mycobacterial cultures identified as non-tuberculous mycobacteria (NTM) (Details are shown in the **Supplementary Materials**).

### 2.2 Bacterial strains

BCG were grown at 37°C in Difco Middlebrook 7H9 broth (Cat: 90003-876, Becton Dickinson) or on Middlebrook 7H10 agar (Cat: 90003-728, Becton Dickinson) supplemented with 10% oleic acid-albumin-dextrose-catalase-enriched Middlebrook (OADC, Cat: 90000-418, Becton Dickinson), 0.2% glycerol, and 0.05% Tween-80. Slow-growing BCG reach logarithmic phase for 3–4 weeks culture. *M. smegmatis* strains grow fast and reach logarithmic phase for 2–3 days culture. BCG and *M. smegmatis* were Collected and stored in PBS containing 10% glycerol at -80°C for long-term preservation (**Table 1**).

**Table 1:** Mycobacteria strains used in the study.

Strains	Purpose
*Mycobacterium bovis*, Bacillus Calmette-Guerin (BCG) Danish strain (ATCC35733)	Infection of cells, exploration of the function and molecular mechanism of miR-107
*Mycobacterium smegmatis* [ATCC00084/mc (2)155]	Detection of expression of miR-107 in exosomes

### 2.3 Cells and cell culture

The human macrophage cell line THP-1 (RRID: CVCL_0006) and human peripheral blood mononuclear cells (PBMC) were cultured in Roswell Park Memorial Institute (RPMI) 1640 medium supplemented with 10% fetal bovine serum (FBS). The human alveolar epithelial cell line A549 (RRID: CVCL_0023) and mouse macrophage RAW264.7 (RRID: CVCL_0493) were maintained in Dulbecco’s Modified Eagle Medium (DMEM) supplemented with 10% FBS. THP-1 cells in concentration of 1×10^6^ per ml were treated with 50 ng/ml phorbol 12-myristate 13-actate (PMA, CAS: 16561-29-8, Sigma-Aldrich) for 48 h to differentiate into macrophages, then washed twice with phosphate buffered saline (PBS) and maintained for further infection or transfection.

### 2.4 Exosome extraction

PMA-treated THP-1 macrophages were transfected with mimics or inhibitors of miR-107 for 4 hours. Then, the medium was replaced with exosome-free RPMI 1640 medium supplemented with 10% FBS (VivaCell). After another 48 hours of incubation, the supernatant was collected for exosome extraction pre-treatment.

PBMCs were isolated from the blood of healthy control and infected with miR-107 overexpressing lentivirus (Tsingke). After 48 hours, the culture medium was collected for exosome extraction pre-treatment.

Fresh plasma from TB and HC donors was spun at 800 g for 10 min to separate plasma from blood cells. The upper plasma layer was then collected for exosome extraction pre-processing.

The collected plasma or supernatant was subjected to a series of centrifugation steps to remove cellular debris and apologetic bodies: 300 g for 10 min at 4°C;3,000 g for 20 min at 4°C;10,000g for 30 min at 4°C. The supernatant was carefully extracted and the resulting pellet discarded. Exosomes were then isolated using the exoRNeasy Exosome Isolation Kit (QIAGEN), following the manufacturer’s instructions, and dissolved in XE Buffer.

### 2.5 RNA extraction and miRNA RT-qPCR assay

RNA was extracted from the following samples for the detection of relevant gene expression: exosomes derived from THP-1, RAW264.7, or PBMCs with either overexpression or knockdown of miR-107; plasma-derived exosomes from HC and TB; THP-1 macrophages with altered miR-107 expression; and PBMCs isolated from HC and TB. RNA was harvested with RNeasy kit (Qiagen) or MiniBEST Universal RNA Extraction Kit (CAT: 9767, TAKARA) as per manufacturer’s protocol. Reverse transcription was performed with PrimeScript™ RT reagent Kit with gDNA Eraser (RR047A, TAKARA) or miRcute Plus miRNA First-Strand cDNA Kit (TIANGEN) as per manufacturer’s instruction. qPCR experiments were performed on an CFX384 Touch Real-Time PCR in 10 μl reactions, using TB Green^®^ Premix Ex Taq™ II (RR820A, TAKARA) for mRNA or miRcute Plus miRNA qPCR Kit (FP411, TIANGEN) for miRNA as per manufacturer’s instructions. The primers used for amplification are listed as following: GAPDH-F: CCTGCCTCTACTGGCGCTGC; GAPDH-R: GCAGTGGGGACACGGAAGGC; miR-107-F: CAGCAGCATTGTACAGGGCTATCA; miR-107-R: GTGCAGGGTCCGAGGT; Wnt16-F: AGGGACACAAGGCAGAGAA T; Wnt16-R: CAACGGACATAGCAGCACC.

### 2.6 Mycobacteria infection of host cells and measurement of intracellular mycobacterial growth

#### 2.6.1 Detection of expression of miR-107 in cells or exosomes after mycobacteria infection

A549 cells (0.5×10^6^ cells/ml) and PMA-treated THP-1 cells (1×10^6^ cells/ml) were infected with BCG at a multiplicity-of-infection (MOI) of 1 bacilli to 1 cell for 4 hours. The cells were then washed three times with PBS to remove any uninternalized BCG and cultured in exosome-free serum medium for 48 hours. After 48 hours, the cell supernatant was collected for exosome extraction, and the cells were lysed for RNA extraction (15).

PBMCs (1.5×10^6^ cells/ml) obtained from HC were cultured in exosome-free serum medium. After infection with *Mycobacterium smegmatis* (MS) for 2 hours or BCG for 4 hours, 2% antibiotics were added to stop the infection. The PBMCs were further cultured for 48 hours, and the supernatant was collected for exosome isolation and subsequent RNA extraction.

#### 2.6.2 Measurement of intracellular mycobacterial growth

In some experiments, PMA-treated THP-1 cells were transfected with miRNA mimics, inhibitors, or respective controls as described above, followed by BCG infection at an MOI of 1 for 4 h. After washing with PBS, the cells were cultured in media without antibiotics for 3 days or the specified time. The supernatant was discarded, and the infected cells were lysed in PBS containing sodium dodecyl sulfate (SDS, Sigma-Aldrich) at a working concentration of 0.3 g/L. Serial dilutions were performed for quantitative culturing, and mycobacterial viability was assessed by colony-forming unit (CFU) counting as previously described (16).

RAW264.7 cells and PMA-treated THP-1 cells were infected with BCG at an MOI of 1 for 4 hours. The cells were washed with PBS and cultured in medium, and exosomes (50μg/ml) enriched with miR-107 mimics or inhibitors were added, followed by incubation for 3 days. The subsequent steps were performed as described above. Notably, quantitative analysis revealed comparable particle concentrations (particles/ml) in plasma-derived exosomal isolates normalized to 50 μg/ml exosomal protein content between TB patients and HC subjects as determined by nanoparticle flow cytometer. This critical normalization step validated the standardized isolation protocol and ensured quantitative parity of exosomal inputs across experimental groups, thereby eliminating particle number variability as a confounding factor in subsequent functional comparisons between cohorts.

PBMCs (1 × 10^7^ cells/ml) obtained from HC were infected with BCG at a MOI of 0.1 for 4 hours. THP-1 cells derived exosomes enriched with miR-107 (50μg/ml) were co-cultured with BCG infected PBMCs for 3 days. Then, SDS was added to lyse the infected cells. The cell lysates were subjected to serial dilution, and aliquots were plated on 7H10 agar plates for CFU counting.

### 2.7 Animal experiments

Female C57BL/6 mice (6–8 weeks old) were challenged via two distinct routes: (1) intranasal instillation of 1×10^6^ CFU BCG in 20 µL PBS or (2) intravenous injection (tail vein) of equivalent BCG CFU. Cardiac blood samples were collected at designated endpoints (day 7, day 11 post-intravenous infection, respectively) and subjected to differential centrifugation (1,000×g, 10 min, 4°C) for plasma isolation. Plasma-derived exosomes were subsequently purified using established protocols, followed by quantification of exosomal miR-107 levels via quantitative real-time PCR (RT-qPCR) as previously described.

In a parallel therapeutic intervention study, BCG-infected mice (intranasal challenge: 1×10^6^ CFU in 20 µL PBS) received intranasal administrations of RAW264.7 macrophage-derived exosomes (5 μg/mouse) at 72-hour intervals (days 3, 7, 10, and 14 post-infection). All animals were humanely euthanized 7 days post-final treatment (day 21). Lung tissues were homogenized in sterile PBS and plated on selective 7H10 agar supplemented with antibiotic cocktail: carbenicillin (50 μg/mL), polymyxin B (33.3 U/mL), amphotericin B (10 μg/mL), and trimethoprim lactate (20 μg/mL). BCG burden was quantified through CFU enumeration after 21-day incubation at 37°C with 5% CO_2_.

### 2.8 miRNA mimics and inhibitors transfection

Cy3-labeled miR-107 mimics (Tsingke), miR-107 mimics (GeneAdvCo), miR-107 inhibitors (GeneAdvCo), and corresponding negative controls (mimic NC and inhibitor NC) were transfected into recipient cells using Lipofectamine™ 2000 reagent (Thermo Fisher Scientific) following the manufacturer’s instructions and our previous studies (17). After 4 hours of transfection, the transfection mixture was replaced with RPMI 1640 medium supplemented with 10% fetal bovine serum (FBS).

### 2.9 Transmission electron microscope detection

Exosomes were first fixed with 2.5% glutaraldehyde at 4 °C overnight. Following washing, the vesicles were deposited onto copper grids coated with a formvar-carbon film, negatively stained with aqueous phosphotungstic acid for 60 seconds, and then visualized with transmission electron microscope (Philips CM-120).

### 2.10 Nanoparticle tracking analysis

Nanoparticle tracking analysis (NTA) was performed using a NanoSight instrument (NanoSight NS300; NTA software version 3.4, Build 3.4.4) to characterize the size profiles of exosomes. Cells were cultured for 48 hours in medium supplemented with 10% exosome-depleted fetal bovine serum. The culture supernatant was collected, and exosomes were isolated and subsequently diluted in ultrapure water for particle size assessment. A particle size distribution (PSD) graph was created with the estimated particle diameter (nm) on the x-axis and the estimated concentration (particles/mL) on the y-axis.

### 2.11 Exosome uptake assay

THP-1 secreted exosomes were incubated with PKH26 for 3 minutes, following the manufacturer’s instructions (Thermo Fisher Scientific). The reaction was terminated by adding an equal volume of exosome-free FBS. Exosomes were then washed twice with PBS. Subsequently, the exosomes (50μg/ml) were co-cultured with THP-1 cells (1×10^6^ cells/ml) in a glass-bottom culture dish (Nest) for 12 hours. After incubation, the cells were washed three times with PBS and fixed in 4% paraformaldehyde (Beyotime) for 20 minutes. Nuclei were stained with DAPI (Beyotime) for 10 minutes, followed by three times of PBS washes. The uptake of exosomes by the cells was observed using a laser scanning confocal microscope.

### 2.12 Co-culture assay

To investigate exosome-mediated miRNA transfer, THP-1 monocytes (1×10^6^ cells/mL) were subjected to a dual-chamber co-culture system using 0.4 μm pore polycarbonate transwell plates (Corning). Donor cells in the upper chamber were transfected with Cy3-labeled miR-107 mimics (50 nM final concentration) as above described, while recipient cells in the lower chamber maintained naive status. Following 12-hour co-culture, recipient cells underwent two PBS washes prior to fluorescence microscopy analysis.

To specifically assess exosome-dependent transfer mechanisms, parallel experiments incorporated pretreatment of donor cells with the neutral sphingomyelinase inhibitor GW4869 (Beyotime Biotechnology, Cat# ST1578; 10 μM) for 24 hours - a validated strategy to block exosome biogenesis. GW4869-pretreated donor cells were subsequently co-cultured with naive recipients under identical conditions. Fluorescence signal quantification was performed using ImageJ software with background subtraction from untreated control wells.

### 2.13 Reactive oxygen species assay

A549 cells (0.5 × 10^4^ cells/ml) were cultured in a 96-well cell culture plate and transfected with miR-107 mimics, inhibitors, or respective controls as described above. After 24 hours, the cells were loaded with probes according to the manufacturer’s instructions, and ROSup (0.5 mg/mL) was added to enhance ROS production. Fluorescence was then measured after 2 hours of co-incubation.

### 2.14 Western blotting assay

WB analysis was employed to identify exosome biomarkers and evaluate the expression of crucial proteins in the Wnt signaling cascade. THP-1 cells transfected with either miR-107 mimics or inhibitors or exosomes were isolated using radioimmunoprecipitation assay (RIPA) buffer (Beyotime) containing a protease inhibitor cocktail (Epizyme) on ice for 10min to harvest proteins. Proteins were separated via sodium dodecyl sulfate-polyacrylamide gel electrophoresis (SDS-PAGE, Epizyme), and then transferred to polyvinylidene fluoride (PVDF) membranes. The membranes were then subjected to blocking with protein-free rapid blocking buffer (1×) (Epizyme) and incubated overnight at 4°C with antibodies against β-catenin (Cell Signaling Technology, 8480), non-phosphorylated (active) β-catenin (Cell Signaling Technology, 8814), c-myc (Cell Signaling Technology, 5605), GAPDH (Cell Signaling Technology, 5174), CD63 (Abcam, ab275018), and Calnexin (Abcam, ab275018). The membranes were thoroughly washed with TBS-T (Beyotime), followed by incubation with peroxidase-conjugated anti-rabbit IgG antibody (Abcam, ab205718) for 1 hour at room temperature. After further washing with TBS-T (Beyotime), protein was detected with ECL reagents (Omni-ECL™ Femto Light Chemiluminescence Kit, Epizyme).

### 2.15 Statistical analysis

All data were tested by the Shapiro-Wilk test to verify the normality. Statistical analysis was performed with GraphPad Prism 9. Differences between groups were assessed using t-test or one-way ANOVA, followed by Tuke’s multiple comparison test, as indicated in the figure.

## 3 Results

### 3.1 MicroRNA miR-107 highly expressed in plasma exosomes but not in PBMC of tuberculosis patients, compared to healthy persons

**Figure 1:**
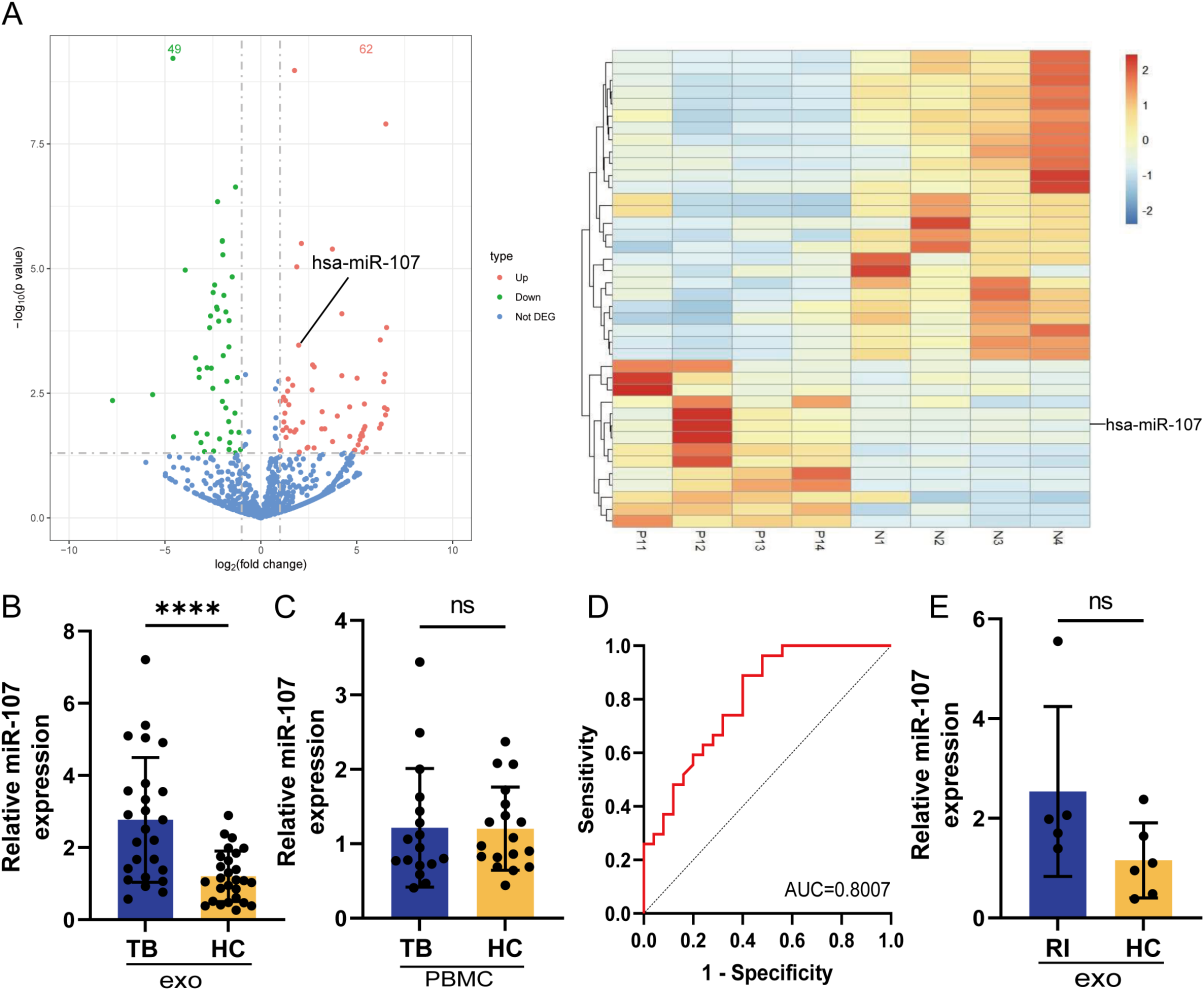
MicroRNA miR-107 highly expressed in plasma exosomes but not in PBMC of tuberculosis (TB) patients, compared to healthy persons. **(A)** Volcano plots showed the difference of microRNA expression profiles in plasma exosomes between TB and healthy control (HC) subjects. There were 62 microRNAs (red) including miR-107 highly expressed, and the expression of 49 microRNAs (green) were down regulated in TB plasma exosomes comparing to those in HC subjects. up, highly expressed miRNAs in TB; down, down regulated miRNAs in TB; Not DEG, miRNAs with no significant differences in expression between TB and HC. The right panel showed the cluster results of the top 40 miRNAs differentially expressed in TB and HC plasma exosomes (n=4). **(B)** The expression of miR-107 was detected by RT-qPCR methods in plasma exosomes from TB patients (n=25) and HC subjects (n=27), respectively. **(C)** The expression of miR-107 in PBMC of TB (n=17) and HC (n=18) were tested by RT-qPCR methods. **(D)** ROC curve analysis for the discriminatory power of plasma exosomes miR-107 for the TB and HC. **(E)** The expression of miR-107 in plasma exosomes of other RI diseases and HC subjects were tested by RT-qPCR. Results are expressed as mean ± SD. ns, not significance; ****p < 0.0001. Statistical significance was determined using Student’s t-test. ROC, receiver operating characteristic; AUC, area under the curve; RI, respiratory infection.

To examine the roles of exosomes in immune responses during Mtb infection, we isolated exosomes from plasma of TB patients and HC subjects and compared their miRNA expression profiles. Our effort engaged the rationale that miRNAs constitute the predominant cargo in exosomes and serve as crucial regulators of protein biosynthesis (18). Our miRNA sequencing results showed that exosomes from TB plasma presented different miRNA expression profiles compared with those in HC plasma (**Figure 1A**). There were 62 miRNAs significantly upregulated (49 miRNA downregulated) in exosomes of TB patients’ plasma, compared to HC subjects (**Figure 1A**). The expression level of microRNA miR-107 was highly elevated in TB plasma exosomes, with approximately 2.5 folds increases when compared to the counterparts of HC controls (**Figure 1B**). This result showed that Mtb infection increased the expression of miR-107 in plasma exosomes. Next, we examined whether miR-107 was also highly expressed in PBMC isolated from TB and HC. Results showed there were no significant difference in miR-107 expression levels between PBMC samples from TB patients and HC subjects (**Figure 1C**). Thus, these experiments demonstrated that miR-107 expressed high in plasma exosomes, but not in PBMC, of TB patients.

We then sought to evaluate whether exosome miR-107 could be used to differentiate TB infection from healthy condition. We performed receiver operating characteristic (ROC) curve analysis. The area under the curve (AUC) value was more than 0.8 (**Figure 1D**), implicating that the expression level of miR-107 in plasma has predictive value to differentiate TB infection and healthy controls. Moreover, we did not detect a high mean level of miR-107 in plasma exosomes isolated from limited numbers of patients with other respiratory infectious diseases rather than TB, with a lack of significant difference in miR-107 expression between patients with other respiratory infectious disease and healthy subjects (**Figure 1E**). Thus, miR-107-high exosomes appeared to be temporally predictive for TB infection in humans.

### 3.2 miR-107-high exosomes were also detected in both the extracellular fluid released by mycobacterial-infected macrophages and the plasma of mycobacterial-infected mice

**Figure 2:**
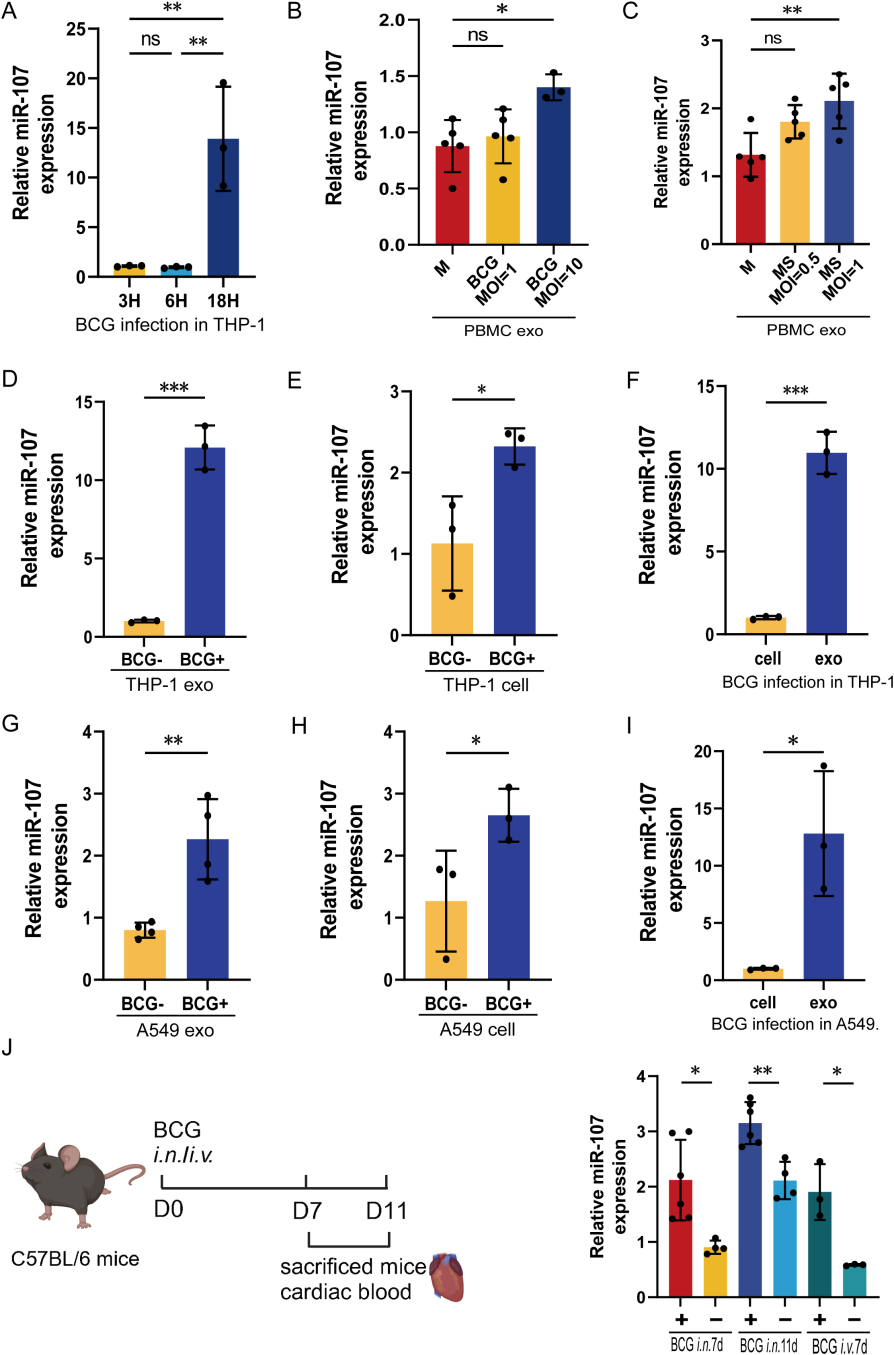
miR-107-high exosomes were detected in both the extracellular fluid released by mycobacterial-infected macrophages and the plasma of mycobacterial-infected mice. **(A)** The expression levels of miR-107 were detected in THP-1 cells after BCG infected at 3 hours (h), 6 h and 18 h, respectively. **(B, C)** The expression levels of miR-107 were tested in exosomes produced by PBMC stimulated with BCG **(B)** or MS **(C)** at different concentrations, respectively. **(D–F)** The expression levels of miR-107 were tested by RT-qPCR in THP-1 cell-derived exosomes **(D)** and THP-1 cells **(E)** with or without BCG infection. **(F)**showed the expression of miR-107 in BCG infected THP-1 and their exosomes. **(G–I)** The expression levels of miR-107 were tested by RT-qPCR in A549 cell-derived exosomes **(G)** and cells **(H)** with or without BCG infection. **(I)** showed the expression of miR-107 in BCG infected A549 and their exosomes. **(J)** The expression levels of miR-107 were detected in plasma exosomes of mice after BCG infection at 7d and 11days, respectively(right). A diagram of experimental procedures (left). Results are expressed as mean ± SD. ns, not significance; *p<0.05, **p<0.01, ***p < 0.001. Statistical significance was determined using Student’s t-test **(D–J)**, one-way ANOVA **(A–C)**. M, medium; MS, *Mycobacterium smegmatis*. Data represent 3 independent experiments.

Since miR-107 highly expressed in exosomes of TB plasma, we then sought to determine whether mycobacterial infection could up-regulate the miR-107 and yield miR-107-high exosomes. We infected THP-1 macrophage with *Mycobacterium bovis* BCG and measured intracellular miR-107 and miR107-high exosomes released by BCG-infected THP-1 cells, respectively. After we optimized the infection conditions, we found the expression levels of miR-107 were significantly increased in 18 hours after BCG infection in THP-1 cells (**Figure 2A**). Moreover, an increased infection dose of mycobacteria led to releasing miR-107-higher exosomes (**Figures 2B, C**).

In fact, mycobacterial BCG infection could induce 12- and 2.1-fold increases in exosome miR-107 and intracellular miR-107, respectively, compared to the controls (**Figures 2D, E**). And the expression level of exosome miR-107 was significantly higher than their intracellular level of BCG-infected THP-1 cells (**Figure 2F**). Similar results were obtained in BCG infected A549 cells (**Figures 2G–I**), which are lung epithelial cells serving also as a target for Mtb infection. Therefore, we demonstrated that mycobacterial BCG infection increased intracellular miR-107, leading to miR-107-higher exosomes released by infected cells. The data were consistent with the earlier results seen in TB patients who exhibited miR-107-higher exosomes in plasma, with no increases in intracellular miR-107 in PBMC.

Next, we did a proof-of-concept experiment to determine the *in vivo* miR-107 expression levels and miR-107-high exosomes in plasma after mycobacterial infection of mice. To this end, mice were infected with *M. bovis* BCG or PBS control, then euthanized at the 7^th^ or 11^th^ day after infection, and then assessed for the miR-107-high exosomes in plasma. The use of BCG here was justified for proving a concept in the acute experimental infection, rather than for inducing chronic TB infection or pathology. Our results showed that both intranasal (i.n.) and intravenous (i.v.) BCG infections significantly increased the expression levels of miR-107-high exosomes in plasma at 7^th^ and 11^th^ days post infection, respectively (**Figure 2J**).

Together, the results above demonstrated that mycobacterial infection induced miR-107-high exosomes both in the extracellular fluid released from infected cells and in the plasma of infected mice, with the exosomes miR-107 being higher than the intracellular levels.

### 3.3 Adding miR-107-high plasma exosomes or miR-107 mimics to infected THP-1 macrophages inhibited intracellular mycobacterial growth

**Figure 3:**
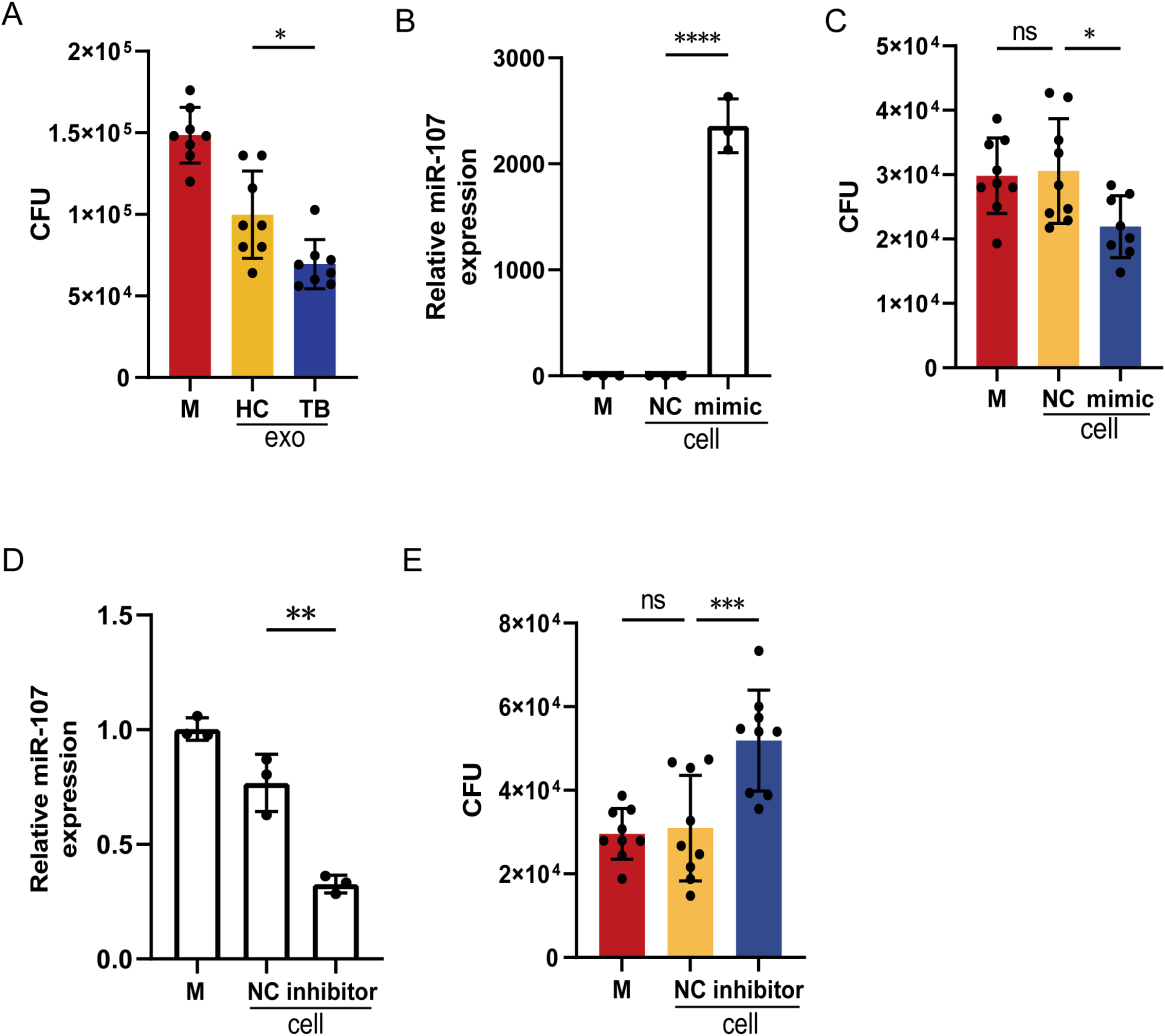
Adding miR-107-high plasma exosomes or miR-107 mimics to infected THP-1 macrophages inhibited intracellular mycobacterial growth. **(A)** The counts of BCG CFU were shown in THP-1 cells treated with plasma exosomes isolated from TB and HC, respectively. Exosomes (50μg/ml) were added into medium of BCG-infected THP-1(n=8) and incubated for 3 days. Then, cells were lysed, and the lysate was diluted to spread on 7H10 plates to count BCG CFU. **(B)** The expression levels of miR-107 were detected in THP-1 cells transfected with miR-107 mimics or controls (NC). **(C)** The BCG CFU were shown in THP-1 cells transfected with miR-107 mimics or controls, respectively. **(D)** The expression levels of miR-107 were detected in THP-1 cells transfected with miR-107 inhibitors or controls (NC). **(E)** The BCG CFU were shown in THP-1 cells transfected with miR-107 inhibitors or controls, respectively. Results are expressed as mean ± SD. ns, not significance; *p<0.05, **p<0.01, ***p < 0.001, ****p < 0.0001. Statistical significance was determined using one-way ANOVA **(A–D)**, Kruskal-Wallis test **(E)**. Data represent 3 independent experiments.

Since exosomes isolated from TB plasma presented different microRNA expression profiles compared to HC subjects, we want to determine whether there was a difference in effector functions between these two sources of plasma exosomes. Thus, we added exosomes isolated from plasma of TB patients and HC subjects to BCG-infected THP-1 cell cultures, respectively. We found that the BCG CFU counts in the cells treated with exosomes isolated from TB patients’ plasma were significantly lower than those from HC subjects (**Figure 3A**). This implied that miR-107-high exosomes isolated from TB plasma possessed significantly potent effector function of inhibiting mycobacteria growth in macrophages.

Since the plasma exosomes of TB patients contained significantly higher levels of miR-107 (**Figures 1A, B**), we wanted to know whether miR-107 in exosomes was indeed an active component that could inhibit intracellular mycobacterial growth. To this end, we molecularly manipulated changes in miR-107 expression levels in BCG-infected macrophages and assessed them for alterations in BCG infection. Virtually, the transfection of miR-107 mimics into THP-1 cells resulted in more than 2000-fold increases in miR-107 expression compared to the controls (**Figure 3B**). Such increased miR-107 mimics in transfected THP-1 cells significantly lowered BCG CFU counts compared to the controls (**Figure 3C**). Concurrently, the transfection of miR-107 inhibitors to THP-1 cells significantly decreased the expression of miR-107 to the level of about 60% of the controls (**Figure 3D**). Such down-regulation of miR-107 expression led to a 1.6-fold increase in BCG CFU counts in THP-1 cells compared to the controls (**Figure 3E**). These results showed that miR-107 and miR-107-high exosomes could potently inhibit mycobacterial growth in the THP-1 macrophages.

### 3.4 miR-107-enriched exosomes potently inhibited intracellular mycobacterial growth in macrophages

**Figure 4:**
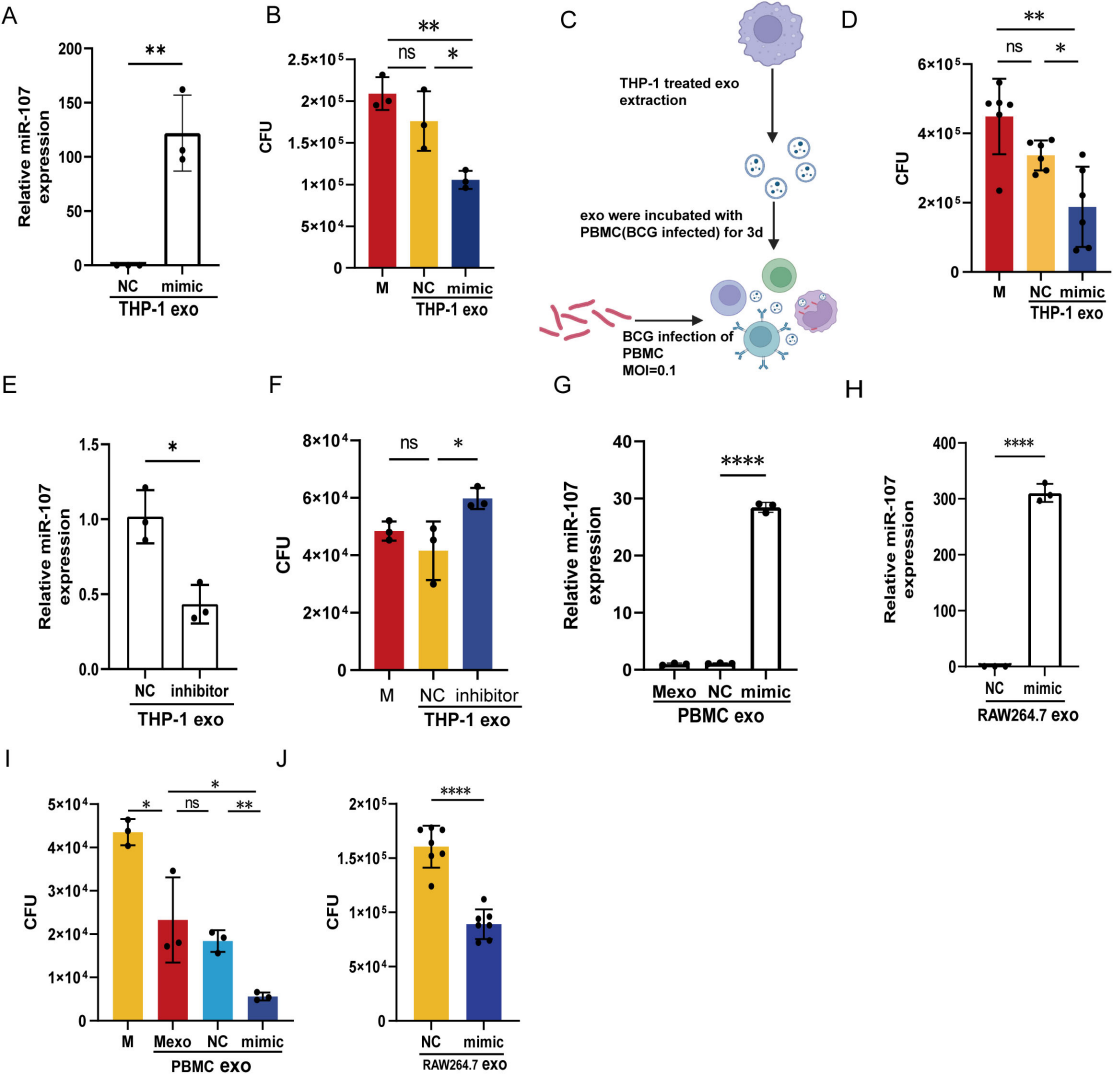
miR-107-enriched exosomes potently inhibited intracellular mycobacterial growth in macrophages. A, **(B)** The expression levels of miR-107 were detected by RT-qPCR in exosomes isolated from THP-1 cells transfected with miR-107 mimics **(A)**. **(B)** Exosomes enriched with miR-107 mimics and their controls (50μg/ml) were added into BCG infected THP-1 cells (MOI=1) and incubated for 3 days, then cells were lysed and used to count BCG CFUs. **(C, D)** Exosomes isolated from THP-1 cells transfected with miR-107 mimics or controls were added into PBMC infected with BCG, and counted BCG CFUs. **(E, F)** The expression levels of miR-107 were detected by RT-qPCR in exosomes isolated from THP-1 cells transfected with miR-107 inhibitors or controls **(E)**, these exosomes were added into THP-1 cells (MOI=1) for 3days and cells were lysed for BCG CFUs counting **(F)**, as mentioned above. **(G–I)** The expression levels of miR-107 were detected by RT-qPCR in exosomes isolated from RAW264.7 cells transfected with miR-107 mimics or controls **(H)**. And, exosomes enriched with miR-107 mimics or controls (50μg/ml) were added into BCG infected RAW264.7 cells (MOI=1), then cells were lysed and used to count BCG CFUs **(J)**. The expression levels of miR-107 were detected by RT-qPCR in exosomes isolated from human PBMC cells infected with lentivirus-mimic **(G)**. The BCG CFUs were counted in BCG infected THP-1 cells (MOI=1) after incubated with exosomes isolated from PBMCs infected with lentivirus overexpressing miR-107 **(I)**. Results are expressed as mean ± SD. ns, not significance; *p<0.05, **p<0.01, ***p < 0.001, ****p < 0.0001. Statistical significance was determined using Student’s t-test **(A, E, H, I, J)**, Mann Whitney test **(D)**, one-way ANOVA **(B, F, G)**. Data represent 3 independent experiments.

Next, we sought to confirm whether miR-107-enriched exosomes consistently inhibited intracellular mycobacterial growth in macrophages. Our experimental system allowed microRNA-overexpressed THP-1 cells to release miR-107-enriched exosomes in which miR-107 expression levels were about 120-fold higher than those from controls (**Figure 4A**). Then, we added these miR-107-enriched exosomes to BCG-infected THP-1 cells, and found that mean BCG CFU counts in cells treated with miR-107-enriched exosomes were reduced down to about 1x10e5 per ml, significantly being lower than those CFU counts 1.7×10e5 per ml as seen in the control (**Figure 4B**). More interesting was that compared with directly elevate increasing the expression of miR-107 within THP-1 cells (28.27%), THP-1 cells treated with exosomes exhibit a stronger capacity to kill BCG (40%). Similarly, these exosomes isolated from miR-107-enriched THP-1 cells could significantly decrease the intracellular BCG growth in primary monocytes/macrophages of human PBMC (**Figures 4C, D**). Consistently, when the expression levels of miR-107 in exosomes isolated from miR-107 inhibitor-transfected THP-1 cells were reduced to the level about half of those in controls (**Figure 4E**), the BCG CFU values in the cells treated with inhibitor-enriched exosomes were increased about 1.5 folds of those in the controls (**Figure 4F**).

Furthermore, we also found a significantly increased miR-107 level in exosomes released from both the human PBMC infected with the miR-107-overexpressing lentivirus (**Figure 4G**) and the mouse macrophages RAW264.7 (**Figure 4H**) transfected miR-107 mimics. Notably, these miR-107-enriched exosomes also potently inhibited intracellular BCG growth in THP-1 (**Figure 4I**) or RAW264.7 (**Figure 4J**) macrophages.

Thus, the above results demonstrated that the exosomes’ miR-107 levels correlated with its intracellular expression in donor cells, and that miR-107-enriched exosomes could inhibit intracellular mycobacterial growth in macrophages.

### 3.5 Nanoscale and fluorescence imaging analyses revealed that miR-107 could be transferred inter-cellularly via exosomes

**Figure 5:**
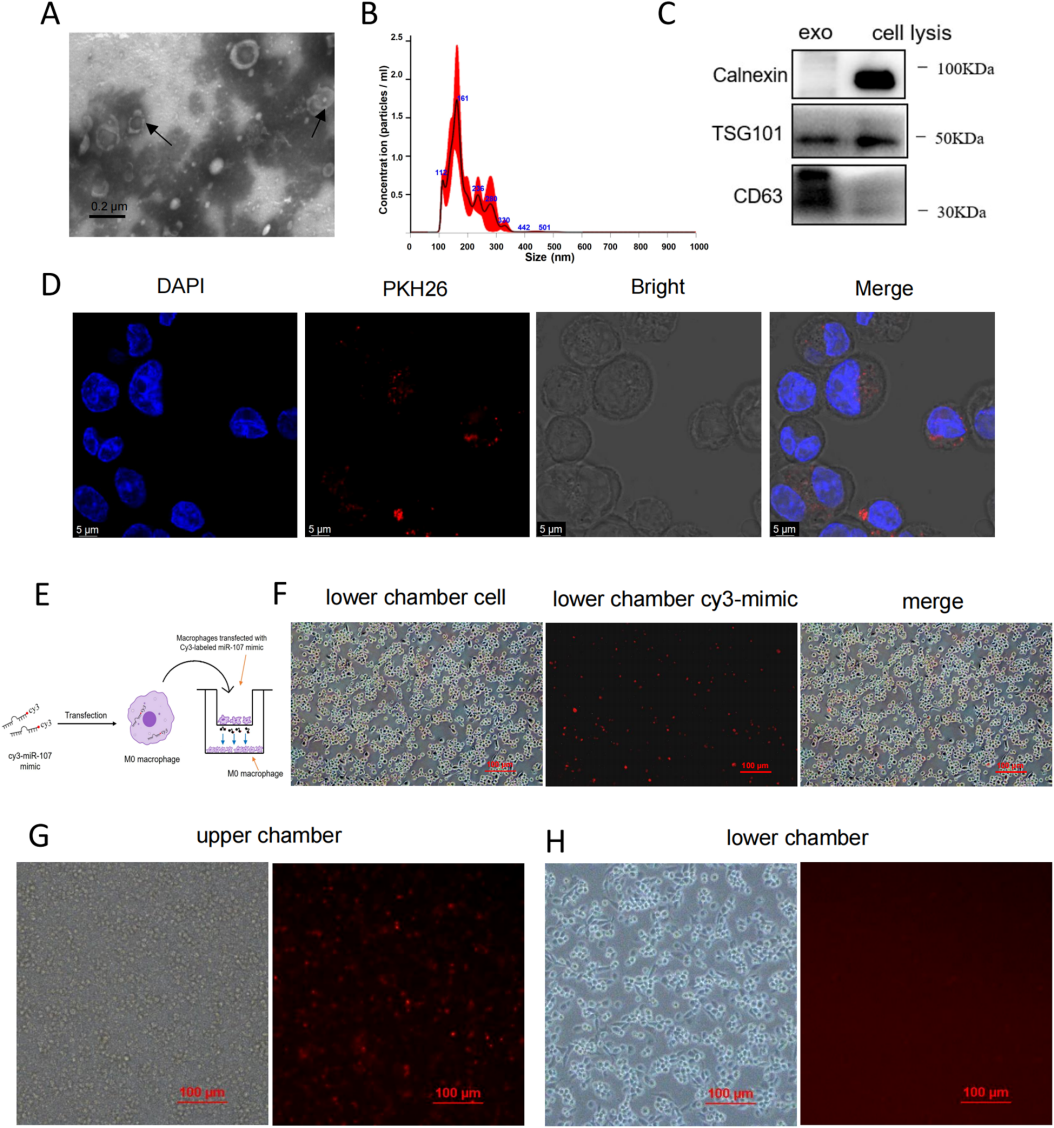
Nanoscale and fluorescence imaging analyses revealed that miR-107 could be transferred inter-cellularly via exosomes. **(A)** Exosomes secreted by THP-1 cells were analyzed under transmission electron microscope (TEM). Scale bar, 0.2μm. **(B)** Nanoparticle tracking analysis (NTA) uncovered the size and concentrations of exosomes. **(C)** Exosome-specific markers TSG101, extracellular vesicle-related protein markers CD63 and proteins calnexin not expressed in exosomes but in cells were measured by western blot analysis. **(D)** MicroRNA miR-107 loaded exosomes were labeled with PKH26 and taken up by THP-1 cells visualized using confocal microscopy. Scale bar, 5μm. **(E)** Diagram of experimental procedures to detect the intercellular transmission of miR-107 labeled with Cy3-fluorescent protein through exosomes. **(F)** Cy3-miR-107 mimics in exosomes of THP-1 cells in the upper chamber were transferred to THP-1 cells in the lower chamber by exosomes. **(G, H)** The effects of the exosomes secretion inhibitor GW4689 (10 mM) on exosome-dependent miRNA delivery from THP-1 cells into THP-1 cells seeded in the lower chamber act as recipient cells. Data represent 3 independent experiments.

To further identify whether miR-107 could transfer into target cells via exosomes, we tracked the uptake of exosomes by recipient cells. In fact, exosomes exhibited cup-shaped or slightly concave discoidal morphology under transmission electron microscope (TEM) (**Figure 5A**). Nanoparticle tracking analysis (NTA) revealed that the majority of exosomes particles constituted a concentration peak with the size 161 nm, while other sub populations of exosomes particles exhibited lower peaks of concentrations ranging from 100 to 236 nm (**Figure 5B**). In addition, immunoblotting analysis showed that the exosomes isolated from THP-1 cells carried TSG101 and CD63 proteins, which are common protein markers of exosomes, with the exosomes lacking the endoplasmic reticulum protein calnexin (**Figure 5C**).

To track the uptake of exosomes by recipient cells, we labeled exosomes with PKH26 and incubated with THP-1 cells, visualization using confocal microscopy showed that PKH26-labeled exosomes were taken up into the cells (**Figure 5D**).

To verify that intracellular overexpressing miR-107 in donor cells could be transferred into other cells via exosomes, miR-107 was labeled with Cy3-fluorescent protein and transfected into THP-1 macrophages cultured in upper chamber of trans-well plate in the presence or absence of exosomes secretion inhibitor GW4689, with un-transfected THP-1 cells placed in lower chamber. After coculture for 12 hours, we found the trans-well occurrence of fluorescence-labeled miR-107 in lower chamber THP-1 cells in the GW4689-absent culture, but not in the GW4689-present culture (**Figures 5E–H**). These visualization results supported the scenario that miR-107 could be transferred via exosomes from donor cells to recipient cells.

### 3.6 MicroRNA miR-107 and miR-107-high exosomes could increase ROS production in macrophages

**Figure 6:**
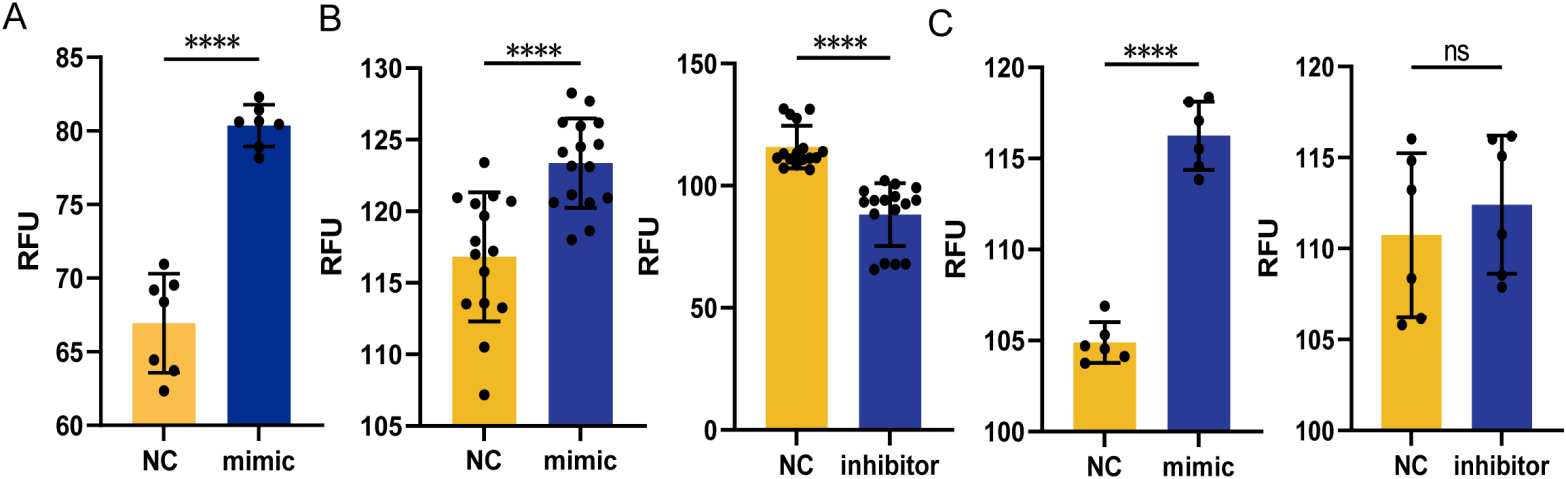
MicroRNA miR-107 and miR-107-high exosomes could increase ROS production in macrophages. **(A)** The production of intracellular ROS was detected in THP-1 cells transfected with miR-107 mimics. **(B)** The production of intracellular ROS was detected in A549 cells transfected with miR-107 mimics (left) or inhibitors (right). **(C)** The production of intracellular ROS was detected in A549 cells incubated with exosomes (50μg/ml) enriched with miR-107 mimics (left) and inhibitors (right) for 12 hours. Results are expressed as mean ± SD. ns, not significance; ****p < 0.0001. Statistical significance was determined using Student’s t-test. Data represent 3 independent experiments.

We already showed that miR-107-enriched exosomes taken-up by recipient cells appeared to inhibit intracellular mycobacterial growth. Here, we would like to investigate a mechanistic action whereby miR-107 regulates the ability of macrophages to inhibit mycobacteria. Since ROS production was reported as one important pathway for macrophages to inhibit intracellular pathogens growth, we elected to measure the production of ROS in THP-1 cells after transfection of miR-107 mimics first (**Figure 6A**). Our results showed that the production of ROS in THP-1 cells transfected with miR-107 mimics were significantly higher than those in the control (**Figure 6A**). Since alveolar epithelial cells also played important roles in anti-tuberculosis immune responses, we also measured the production of ROS in A549 cells transfected with miR-107 mimics or inhibitors and their counterpart controls, respectively. Our results showed that the production of ROS in A549 cells transfected with miR-107 mimics were significantly higher than those in the control (**Figure 6B** left). Consistently, the ROS production in A549 cells transfected with miR-107 inhibitors was obviously lower than the control (**Figure 6B** right). Furthermore, we found that the ROS production in A549 cells cocultured with miR-107-enriched exosomes from miR-107 mimics-transfected THP-1 cells was significantly higher than cells with the controls (**Figure 6C** left). Thus, these results suggested that the increased production of ROS by miR-107 or miR-107-enriched exosomes might help explain their ability to inhibit intracellular mycobacteria.

### 3.7 MicroRNA miR-107 regulated Wnt pathway by targeting Wnt16 and promoted autophagy

**Figure 7:**
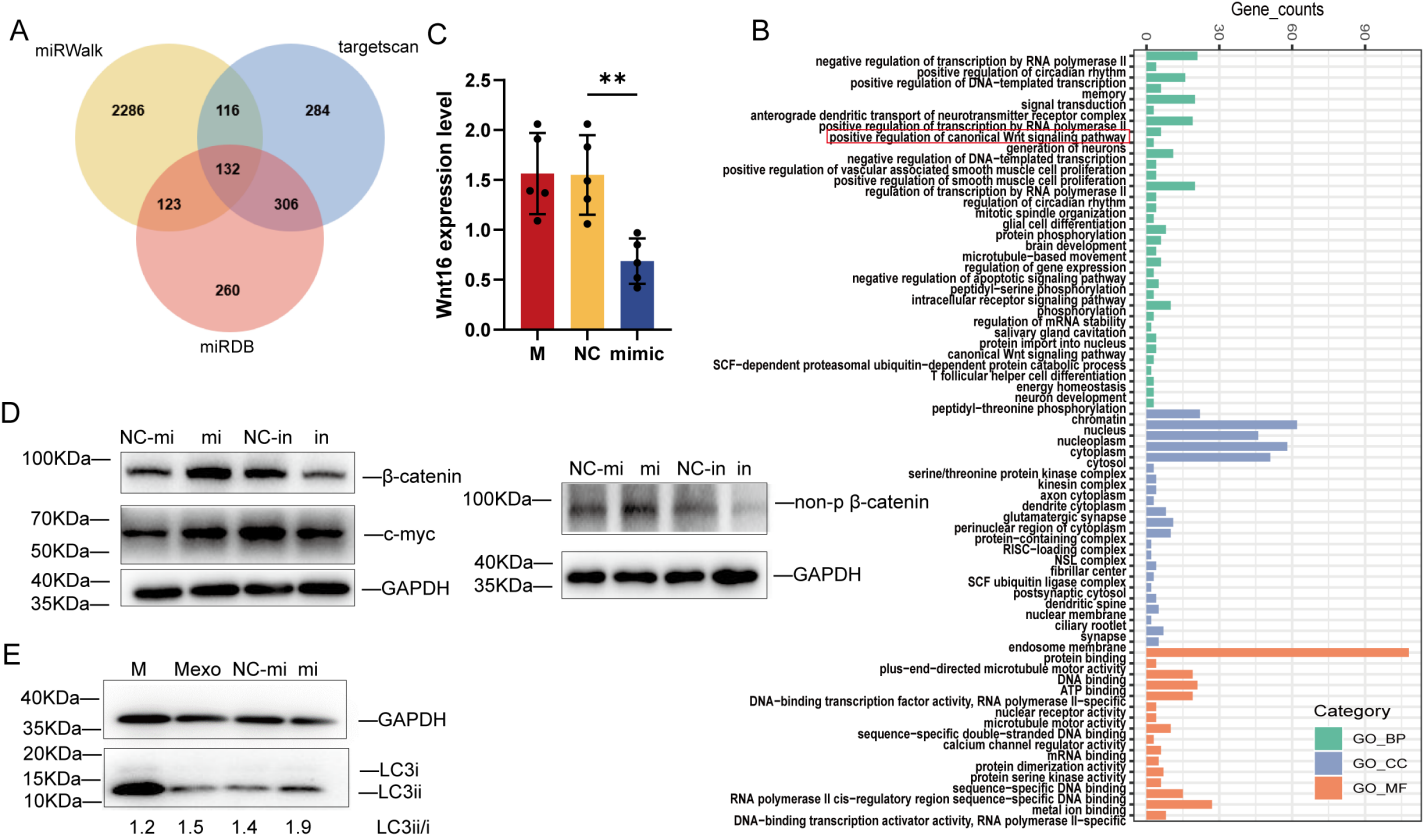
MicroRNA miR-107 regulated Wnt pathway by targeting Wnt16 and promoted autophagy. **(A)** Venn diagram was used to depict the number of potential targets of miR-107 predicted by three algorithms. **(B)** GO pathway enrichment analysis was performed for potential target genes of miR-107. **(C)** Relative expression of Wnt16 was detected in A549 cells after transfection with miR-107 mimics. **(D)** The expression of β-catenin/non-p β-catenin and c-myc of the Wnt pathway were detected by Western blot in PMA-treated THP-1 cells transfected with miR-107 mimics or inhibitors. **(E)** Expression of LC3ii/LC3i in the autophagy pathway was detected by Western blot in PMA-treated THP-1 cells pre-incubated with control or exosomes containing miR-107 mimics. Results are expressed as mean ± SD. **p < 0.01. Statistical significance was determined using one-way ANOVA. Data represent 3 independent experiments.

To identify the microRNA mechanism specific for miR-107, we examined the predicted genes targeted by miR-107 using the three sources: miRWalk, targetscan and miRDB (19). There were 132 common genes among three sources (**Figure 7A**). We performed GO pathway enrichment analysis, and found that some common genes were enriched in “positive regulation of canonical Wnt signaling pathway” including the gene of Wnt16 (**Figure 7B**). To further verify the miR-107 regulatory effect on Wnt16, we measured the expression levels of Wnt16 in A549 cells transfected with miR-107 mimics. Results showed that the expression levels of Wnt16 were significantly decreased in miR-107-enriched A549 cells compared to the controls (**Figure 7C**). These results implied that miR-107 could regulate Wnt16 expression.

Since Wnt16 was reported to negatively regulate canonical wnt pathway (20), we decided to measure the expression of key marker proteins of wnt pathway in THP-1 cells transfected with miR-107 mimics or inhibitors and their counterpart controls. Our immunoblotting analysis showed that the expression of β-catenin and c-myc was increased in miR-107 mimics enriched cells, but was decreased in miR-107 inhibitors transfected cells (**Figure 7D** left). Given that β-catenin degradation is initiated upon amino-terminal serine/threonine phosphorylation, we also measured the expression of non-phosphorylated β-catenin, which is an active form of the protein. Our results showed that the expression of non-phosphorylated β-catenin was increased in miR-107 mimics transfected cells (**Figure 7D** right). These results demonstrated that expression of miR-107 activated wnt pathway by targeting Wnt16.

Furthermore, we found that miR-107-enriched exosomes could enhance autophagy of THP-1 cells as the expression of ILC3II protein was significantly increased (**Figure 7E**). Thus, our results are consistent with the publications which indicate that promoting ROS, Wnt or autophagy pathway can inhibit intracellular mycobacterial infections (21, 22).

### 3.8 Treatment of infected mice with miR-107-enriched exosomes significantly reduced mycobacterial infection in lung tissue

**Figure 8:**
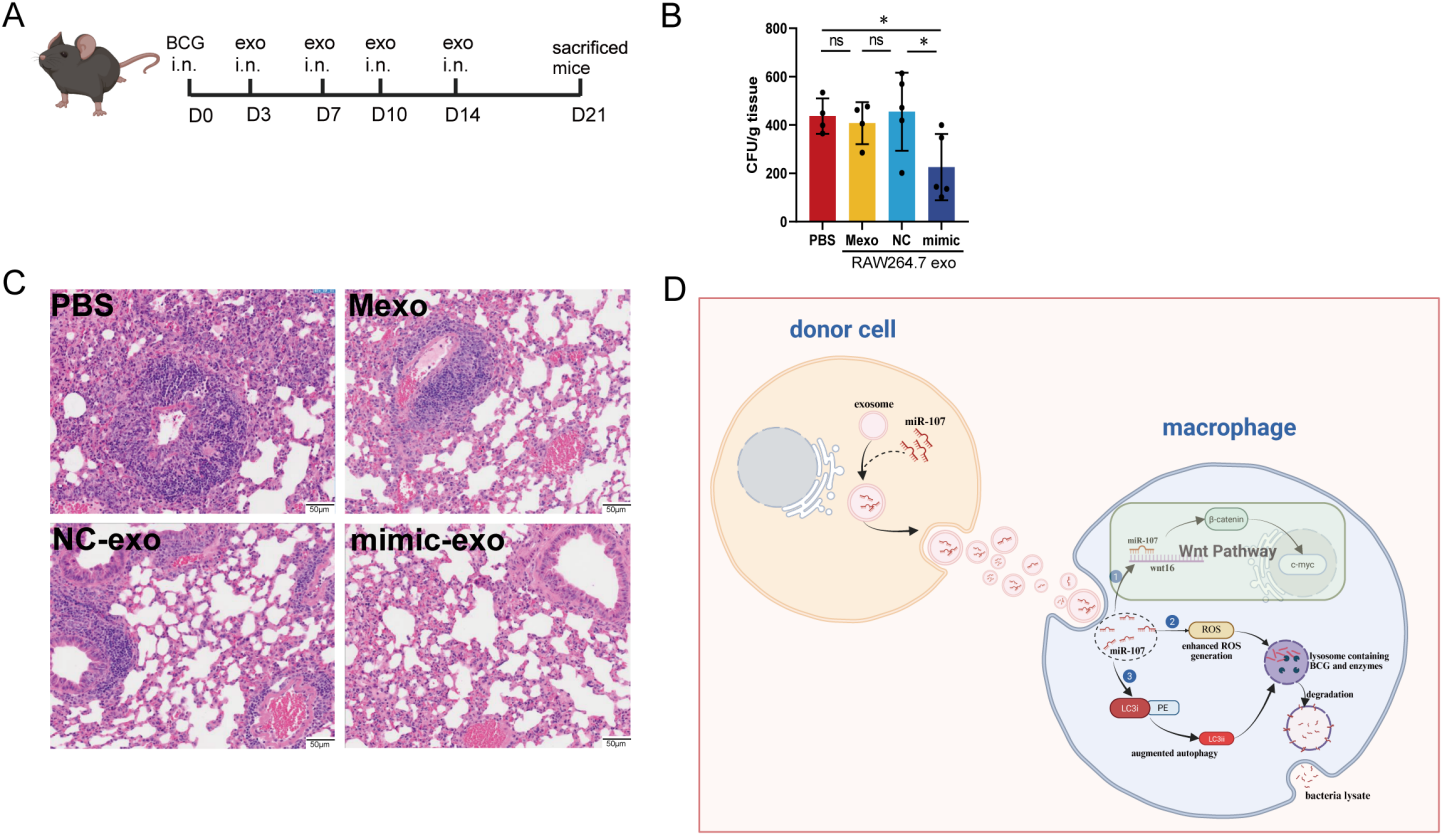
Treatment of infected mice with miR-107-enriched exosomes significantly reduced mycobacterial infection in lung tissues. **(A)** Diagram of the animal experimental procedures. Mice were challenged with mycobacteria BCG, then were treated with exosomes at 3^rd^ day, 7^th^ day, 10^th^ day and 14^th^ day after infection. At 21^th^ day, mice were sacrificed. **(B)** The BCG CFUs in mice lungs were counted at 21^th^ days after BCG challenge (n = 5). **(C)** Showed the representative histological images of H&E-stained mouse lung sections. Results are expressed as mean ± SD. ns, not significance; *p < 0.05. Statistical significance was determined using Student’s t-test. **(D)** The graphic is a hypothetical schematic model. MicroRNA miR-107 could be transferred from donor cells into recipient cells through exosomes, and miR-107 enriched exosomes potently inhibited mycobacteria growth in macrophages through ROS, Wnt and autophagy pathways. Data represent 3 independent experiments.

We already showed that miR-107-enriched exosomes could potently inhibit intracellular mycobacterial growth *in vitro*. These results prompted us to examine whether treatment with miR-107-enriched exosomes could inhibit mycobacterial infection *in vivo*. Here we conducted a proof-of-concept study in BCG-infected mice. The acute use of BSL-2 mycobacterial BCG appeared to be justified, because we focused on primary mycobacterial infection levels, rather than chronic pathogenesis or pathology of tuberculosis. Thus, mice were infected with BCG in nasal drip and treated at days 3, 7, 10 and 14 with miR-107-enriched exosomes, which were isolated from miR-107 mimics-transfected RAW264.7 macrophages (**Figure 8A**). PBS and exosomes from untreated RAW264.7 cells (Mexo) served as controls. Interestingly, we found that the mice treated with miR-107-enrichced exosomes exhibited significantly lower BCG CFU counts in lung tissues at the end point day 21comparied to the controls (**Figure 8B**). Moreover, histology analysis in lung sections showed that the mice treated with miR-107-enriched exosomes displayed much less degrees of acute infection-driven changes characterized by exudates and infiltration of inflammatory cells in lung tissues compared to the control groups (**Figure 8C**). Thus, treatment of infected mice with miR-107-enriched exosomes could potently reduce mycobacterial infection in in lung tissues.

## 4 Discussions

In this study, we found that miR-107 was highly expressed in exosomes isolated from plasma of TB patients compared to healthy subjects. In addition, miR-107-high exosomes were also detected in both the extracellular fluid released by mycobacterial-infected macrophages and the plasma of mycobacterial-infected mice. Notably, up-regulated miR-107 in THP-1 cells led to an increase in intracellular ROS level which played a critical role in inhibiting mycobacterial growth in macrophages. Moreover, while miR-107 or miR-107 exosomes up-regulated Wnt and autophagy pathways, and miR-107-enriched exosomes could also potently inhibit mycobacterial growth in alveolar epithelial cells, which were also important host cells of Mtb infection extensively used *in vitro* studies (23). These results are consistent with the published data indicating that activating of ROS, Wnt or autophagy can inhibit intracellular mycobacterial infection (24, 25). Furthermore, treatment of mycobacterial-infected mice with miR-107-enriched exosomes reduced infection levels and infection-induced changes in lung tissues.

Our results also suggest that miR-107-high exosomes in plasma could potentially serve as a surrogate marker for TB infection in humans. This notion was supported by our observation demonstrating that miR-107-high exosomes were detected in the plasma of TB patients, but not in healthy control. In addition, in a limited number of preliminary tests involving patients with other respiratory infectious diseases (excluding TB), we found that there was no significant difference in miR-107 expression levels in exosomes isolated from these patients’ plasma comparing to healthy control subjects. However, previous studies have reported altered expression levels of miR-103/107 in the plasma of patients with sepsis-associated ARDS (26). These results in preliminary experiments raise a possibility that larger numbers of patients/subjects should be recruited for conclusive and in-depth studies to explore the miR-107-high exosomes for diagnosis of TB infection in combination with other markers (27).

Interestingly, miR-107 was highly expressed in exosomes isolated from plasma of TB patients, whereas there was no significant difference in miR-107 expression in PBMCs between TB patients and HC. It is likely that miR-107-high exosomes are released to plasma from Mtb-infected macrophages/epithelial cells in lungs of TB patients. This statement is supported by our mechanistic miR-107 transfer experiments as well as infection studies both *in vitro* and *in vivo*. Our infection and imaging experiments showed that nanoscale miR-107 exosomes, while secreting them extracellular/inter-cellularly, appeared to transfer intracellular miR-107 to other cells via exosomes transmembrane action (**Figure 8D**). Transfection of miR-107 mimics into macrophages could lead to extracellular releasing of miR-107-high exosomes. Furthermore, even nasal delivery of BCG for lung infection could lead to miR-107-high exosomes in plasma.

Data from the current study also implicate that exosomes enriched with miR-107 or other bio-active molecules may potentially serve as an attractive approach for treatment of infection. Treatment of infected THP-1 macrophages with miR-107-enriched exosomes from microRNA mimic-treated macrophages could readily inhibit mycobacterial growth (**Figure 8D**). Consistently, treatment of infected mice with such miR-107-enriched exosomes also significantly reduced mycobacterial lung infection. These experimental data appear to uncover two meaningful aspects: (i) use of exosomes may avoid administering of huge-doses of miR-107 for inducing anti-mycobacterium effects. In fact, we and others previously showed that high doses of miRNA were required to see miRNA-mediated effects (28, 29); (ii) intranasal administration of exosomes appeared to be effective for delivering miR-107 to lungs and achieving detectable therapeutics. Future fine-tuning studies may provide insights into innovative use of exosomes for therapeutics.

## Data Availability

The original contributions presented in the study are included in the article/**Supplementary Material**, further inquiries can be directed to the corresponding author/s.
