# Supplementary material for: miR-107-enriched exosomes promote ROS/wnt/autophagy, inhibit intracellular mycobacterial growth and attenuate lung infection
Source: Front Immunol. 2025 Jul 4;16:1567167. doi: 10.3389/fimmu.2025.1567167 (PMC12271158; doi:10.3389/fimmu.2025.1567167)
Supplement: Supplementary file 1 [file DataSheet1.pdf]

Supplementary Material

Table 1. Demographic Characteristics of Study Participants

characteristic	Patients With TB	Healthy Controls
Age,y,mean±SD	55.61±14.27	52.7±13.91
Sex		
Female	22	34
Male	32	48
T-SPOT.TB test result		
Positive	54	0
Negative	0	82
Chest radiographic screening		
Positive	54	0
Negative	0	82
Pulmonary tuberculosis	54	N/A*
Treament time < 1 week	54	N/A

*Not applicable

ID	Sex	Age (years)
TB-01	Female	67
TB-02	Female	52
TB-03	Male	55
TB-04	Female	69
TB-05	Male	53
TB-06	Male	62
TB-07	Male	72
TB-08	Female	67
TB-09	Male	62
TB-10	Male	68
TB-11	Female	54
TB-12	Male	55
TB-13	Male	44
TB-14	Male	54
TB-15	Male	63
TB-16	Male	53
TB-17	Female	29
TB-18	Male	72
TB-19	Male	56
TB-20	Female	58
TB-21	Male	38
TB-22	Male	25
TB-23	Female	62
TB-24	Male	58
TB-25	Male	31
TB-26	Male	60
TB-27	Female	59
TB-28	Female	31
TB-29	Male	35
TB-30	Female	29
TB-31	Female	76
TB-32	Female	56
TB-33	Male	44
TB-34	Male	63
TB-35	Female	65
TB-36	Male	66
TB-37	Male	57
TB-38	Male	78
TB-39	Male	68

**TB-40**	Female	**73**
**TB-41**	Female	**33**
**TB-42**	Female	**69**
**TB-43**	Female	**59**
**TB-44**	Male	**27**
**TB-45**	Female	**40**
**TB-46**	Male	**35**
**TB-47**	Male	**65**
**TB-48**	Female	**51**
**TB-49**	Male	**78**
**TB-50**	Male	**65**
**TB-51**	Male	**49**
**TB-52**	Female	**67**
**TB-53**	Female	**74**
**TB-54**	Male	**52**
**HC-01**	Male	**66**
**HC-02**	Female	**52**
**HC-03**	Female	**38**
**HC-04**	Male	**56**
**HC-05**	Male	**58**
**HC-06**	Male	**44**
**HC-07**	Male	**48**
**HC-08**	Male	**64**
**HC-09**	Male	**58**
**HC-10**	Male	**44**
**HC-11**	Male	**58**
**HC-12**	Male	**47**
**HC-13**	Male	**28**
**HC-14**	Female	**43**
**HC-15**	Female	**54**
**HC-16**	Male	**56**
**HC-17**	Female	**29**
**HC-18**	Male	**35**
**HC-19**	Female	**65**
**HC-20**	Female	**59**
**HC-21**	Male	**62**
**HC-22**	Male	**41**
**HC-23**	Male	**36**
**HC-24**	Male	**26**
**HC-25**	Female	**60**
**HC-26**	Male	**60**
**HC-27**	Male	**75**
**HC-28**	Female	**52**

**HC-29**	Female	**53**
**HC-30**	Male	**30**
**HC-31**	Male	**64**
**HC-32**	Male	**49**
**HC-33**	Male	**59**
**HC-34**	Female	**66**
**HC-35**	Female	**74**
**HC-36**	Female	**47**
**HC-37**	Male	**53**
**HC-38**	Female	**65**
**HC-39**	Female	**33**
**HC-40**	Male	**43**
**HC-41**	Male	**54**
**HC-42**	Male	**54**
**HC-43**	Female	**48**
**HC-44**	Female	**57**
**HC-45**	Male	**57**
**HC-46**	Female	**31**
**HC-47**	Female	**66**
**HC-48**	Male	**71**
**HC-49**	Female	**56**
**HC-50**	Male	**32**
**HC-51**	Male	**67**
**HC-52**	Male	**38**
**HC-53**	Female	**46**
**HC-54**	Male	**68**
**HC-55**	Female	**63**
**HC-56**	Female	**46**
**HC-57**	Female	**73**
**HC-58**	Male	**50**
**HC-59**	Male	**61**
**HC-60**	Male	**69**
**HC-61**	Female	**24**
**HC-62**	Female	**67**
**HC-63**	Male	**73**
**HC-64**	Male	**68**
**HC-65**	Male	**58**
**HC-66**	Female	**45**
**HC-67**	Female	**68**
**HC-68**	Female	**35**
**HC-69**	Male	**71**
**HC-70**	Male	**21**
**HC-71**	Male	**59**
**HC-72**	Male	**72**

**HC-73**	Female	**62**
**HC-74**	Male	**23**
**HC-75**	Female	**62**
**HC-76**	Male	**49**
**HC-77**	Male	**62**
**HC-78**	Female	**63**
**HC-79**	Male	**66**
**HC-80**	Female	**30**
**HC-81**	Male	**52**
**HC-82**	Female	**34**
